# Supplementary material for: High Prevalence of Energy and Nutrients Inadequacy among Brazilian Older Adults
Source: Nutrients. 2023 Jul 21;15(14):3246. doi: 10.3390/nu15143246 (PMC10384757; doi:10.3390/nu15143246)
Supplement: Supplementary file 1 [file nutrients-15-03246-s001.zip › nutrients-2461118-supplementary.pdf]

nutrients

Supplementary Table S1. Prevalence of nutrients intake inadequacies among older adults according to sarcopenia diagnosis.

Nutrients	Category	Non-Sarcopenic	Sarcopenic	p-value[1]
Energy (kcal)	Inadequacy	227 (98.7 %)	63 (96.9 %)	0.304
	Adequacy	3 (1.3 %)	2 (3.1 %)	
Protein (g/kg)	Inadequacy	152 (66.1 %)	49 (75.4 %)	0.176
	Adequacy	78 (33.9 %)	16 (24.6 %)	
Dietary fiber (g)	Inadequacy	223 (97.0 %)	63 (96.9 %)	1.000
	Adequacy	7 (3.0 %)	2 (3.1 %)	
Vitamin A (ug)	Inadequacy	107 (46.5 %)	32 (49.2 5)	0.779
	Adequacy	123 (53.5 %)	33 (50.8 %)	
Vitamin B1 (mg)	Inadequacy	276(11.3 %)	12 (18.5 %)	0.143
	Adequacy	204 (88.7 %)	53 (81.5 %)	
Vitamin B2 (mg)	Inadequacy	80 (34.8 %)	18 (27.7 %)	0.301
	Adequacy	150 (65,2 %)	47 (72,3 %)	
Vitamin B3 (mg)	Inadequacy	75 (32.6 %)	22 (33.8 %)	0.882
	Adequacy	155 (67.4 %)	43 (66.2 %)	
Vitamin B6 (mg)	Inadequacy	170 (73.9 %)	55 (84.6 %)	0.098
	Adequacy	60 (26.1 %)	10 (15.4 %)	
Vitamin B12 (ug)	Inadequacy	161 (70.0)	48 (73.8 %)	0.643
	Adequacy	69 (30.0 %)	17 (26.2 %)	
Vitamin D (ug)	Inadequacy	222 (96.5 %)	61 (93.8 %)	0.306
	Adequacy	8 (3.5 %)	4 (6.2 %)	
Vitamin E (mg)	Inadequacy	187 (81.3%)	56 (86.2 %)	0.462
	Adequacy	43 (18.7 %)	9 (13.8 %)	
Copper (ug)	Inadequacy	230 (100.0 %)	65 (100.0 %)	NA [2]
	Adequacy	0 (0.0 %)	0 (0.0 %)	
Phosphorus (mg)	Inadequacy	123 (53.5 %)	40 (61.5 %)	0.262
	Adequacy	107 (46.7 %)	25 (38.5 %)	
Magnesium (mg)	Inadequacy	227 (98.7 %)	65 (100.0 %)	1.000
	Adequacy	3 (1.3 %)	0 (0.0 %)	
Potassium (g)	Inadequacy	224 (97.4 %)	65 (100.0 %)	0.345
	Adequacy	6 (2.6 %)	0 (0.0 %)	
Selenium (ug)	Inadequacy	131 (57.0 %)	38 (58.5 %)	0.888
	Adequacy	99 (43.0 %)	27 (41.5 %)	
Zinc (mg)	Inadequacy	73 (31.7%)	17 (26.2 %)	0.447
	Adequacy	157 (68.3 %)	48 (73.8 %)	
Calcium (mg)	Inadequacy	228 (99.1 %)	63 (96.9 %)	0.212
	Adequacy	2 (0.9 %)	2 (3.1 %)	
Iron (mg)	Inadequacy	51 (22.2 %)	19 (29.2 %)	0.250
	Adequacy	179 (77.8 %)	46 (70.8 %)	

[1] Fisher exact test. [2] NA = Not applicable.
